# Electropositive Citric Acid-Polyethyleneimine Carbon Dots Carrying the PINK1 Gene Regulate ATP-Related Metabolic Dysfunction in APP/PS1-N2a Cells

**DOI:** 10.3390/molecules29091907

**Published:** 2024-04-23

**Authors:** Si Yu, Feng Guo, Yuzhen Luo, Xingfang Zhang, Chenyu Wang, Yiheng Liu, Haiying Zhang

**Affiliations:** 1 Key Laboratory of Brain Science and Health Translational Medicine Research Center in Tropical Environment of Hainan Province, Hainan Medical University, Haikou 571199, China; mytdhkxa@163.com (S.Y.); guofeng_jane@163.com (F.G.); luoyz3190@163.com (Y.L.); 15511050106@163.com (X.Z.); 2 Clinical Medical College, Gannan Medical University, Ganzhou 341000, China; wangchenyu@gmu.edu.cn; 3 Affiliated Haikou Hospital of Xiangya Medical College, Central South University, Haikou 571199, China; liuyiheng2020@126.com; 4 Hainan Provincial Key Laboratory of Carcinogenesis and Intervention, Hainan Medical University, Haikou 571199, China

**Keywords:** Alzheimer’s disease, PINK1 gene, electron transport chain, ATP-related metabolomics

## Abstract

(1) Background: Alzheimer’s disease (AD) is characterized by β-amyloid (Aβ) peptide accumulation and mitochondrial dysfunction during the early stage of disease. PINK1 regulates the balance between mitochondrial homeostasis and bioenergy supply and demand via the PINK1/Parkin pathway, Na^+^/Ca^2+^ exchange, and other pathways. (2) Methods: In this study, we synthesized positively charged carbon dots (CA-PEI CDs) using citric acid (CA) and polyethyleneimine (PEI) and used them as vectors to express PINK1 genes in the APP/PS1-N2a cell line to determine mitochondrial function, electron transport chain (ETC) activity, and ATP-related metabolomics. (3) Results: Our findings showed that the CA-PEI CDs exhibit the characteristics of photoluminescence, low toxicity, and concentrated DNA. They are ideal biological carriers for gene delivery. PINK1 overexpression significantly increased the mitochondrial membrane potential in APP/PS1-N2a cells and reduced reactive-oxygen-species generation and Aβ1-40 and Aβ1-42 levels. An increase in the activity of NADH ubiquinone oxidoreductase (complex I, CI) and cytochrome C oxidase (complex IV, CIV) induces the oxidative phosphorylation of mitochondria, increasing ATP generation. (4) Conclusions: These findings indicate that the PINK gene can alleviate AD by increasing bioenergetic metabolism, reducing Aβ1-40 and Aβ1-42, and increasing ATP production.

## 1. Introduction

Alzheimer’s disease (AD) is a chronic neurodegenerative disorder characterized by the progressive and selective loss of neurons, resulting in memory impairment and cognitive performance deficits [1,2]. Mitochondria in neurons are crucial for regulating neurotransmitter release, neurogenesis, and neural plasticity. Moreover, ATP and tricarboxylic-acid (TCA) cycle intermediates are provided by mitochondria for the production of glutamate and gamma-aminobutyric acid neurotransmitters [3]. At present, the treatment of AD is still based on drug therapy, including cholinesterase inhibitors and N-methyl-D-aspartic acid (NMDA) antagonists [4]. Treatments that interfere with the Aβ generation pathway are also being actively studied. In June 2021, aducanumab was first approved for the treatment of AD in the United States. Aducanumab is a human immunoglobulin γ1 monoclonal antibody that selectively removes aggregated soluble or insoluble Aβ [5]. In January 2023, another monoclonal antibody, lecanemab, was also approved for the treatment of AD [6]. However, aducanumab and lecanemab still cannot hinder the decline of cognitive function in patients [7]. Therefore, the development of AD therapeutic drugs is necessary.

Previous studies have shown that mitochondrial dysfunction and oxidative stress, which decrease lipid peroxidation, increase reactive oxygen species (ROS), decrease metabolism, and promote apoptosis, are early events in AD pathogenesis [8,9]. Therefore, there is a complex relationship between AD pathogenesis and mitochondrial damage, and targeting mitochondria could thus be a major component of AD treatment.

PTEN-induced kinase 1 (PINK1) regulates mitochondrial biogenesis, mitochondrial dynamics, and mitophagy through a variety of pathways and is essential for mitochondrial homeostasis [10,11]. Normal mitochondria have stable energy metabolism and quality control to maintain normal neuronal and synaptic function, while the imbalance of mitochondrial homeostasis in neurons in AD occurs before Aβ deposition and neurofibrillary tangle formation [12]. Studies have shown that β-amyloid (Aβ) deposition, mitochondrial abnormalities, learning and memory impairment, and decreased synaptic plasticity occur in the brains of PINK1-deficient mAPP mice [13]. Overexpression of PINK1 in an AD model inhibited mitochondrial fusion, improved mitochondrial dysfunction, and reduced the production of neuroinflammatory cytokines [14]. Therefore, PINK1-mediated multiple-life activities can improve AD-related pathology by regulating mitochondrial homeostasis.

The high metabolic activity of neurons highlights their high dependence on ATP. An individual resting cortical neuron uses approximately 4.7 billion ATP/s [15]. The consequences of decreased ATP production are extensive. A decrease in ATP levels can attenuate the ability of neurons to maintain an ion gradient and inhibit the generation and propagation of action potentials, thereby impeding nerve transmission [16]. Furthermore, synapses are more prone to dysfunction and degeneration [17]. In AD, the lack of energy produced by neuronal glucose and mitochondria can damage the clearance of intracerebral Aβ1-42 and hyperphosphorylated tau proteins. Aβ1-42 and hyperphosphorylated tau protein deposition can damage mitochondria, decrease energy production, and increase oxidative stress [18].

The emergence of nanoparticles provides a novel strategy for medicinal material delivery. Carbon dots (CDs) are candidate nanomaterials that can cross the blood-brain barrier (BBB) due to their unique characteristics, such as nanoscale size, great biocompatibility, surface modifiability, and photoluminescence [19]. The poor targeting and permeability of drugs for AD treatment are due to the limitations of the BBB [20]. Nevertheless, previous studies have shown that glucose-generated CDs pass through the BBB through glucose transporters [21]. CDs can be combined with memantine hydrochloride through amidation reactions, and nanoscale CDs can be used to transport memantine hydrochloride to penetrate the BBB and inhibit the aggregation of the tau protein [22]. Taken together, these findings indicate that CDs have excellent delivery ability and are among the most promising drug delivery systems.

Under non-stress conditions, PINK1 is cleaved and degraded in mitochondria or lysosomes. Furthermore, only a small amount of PINK1 remains in the cell and cannot aggregate or be activated [23]. In this study, we transfected APP/PS1-N2a cells with CDs carrying the PINK1 gene to determine the protective effect of PINK1 on APP/PS1-N2a cells. The PINK1 gene was combined with CDs and transfected into APP/PS1-N2a cells. After 24 h of transfection, changes in the mitochondrial membrane potential (MMP), ROS, and Aβ1-40 and Aβ1-42 levels were detected. We then investigated the potential effect of PINK1 on mitochondrial energy metabolism by detecting mitochondrial respiratory-chain complex activities and ATP-related metabolite levels.

## 2. Results

### 2.1. Characterization of the CA-PEI CDs

**Figure 1:**
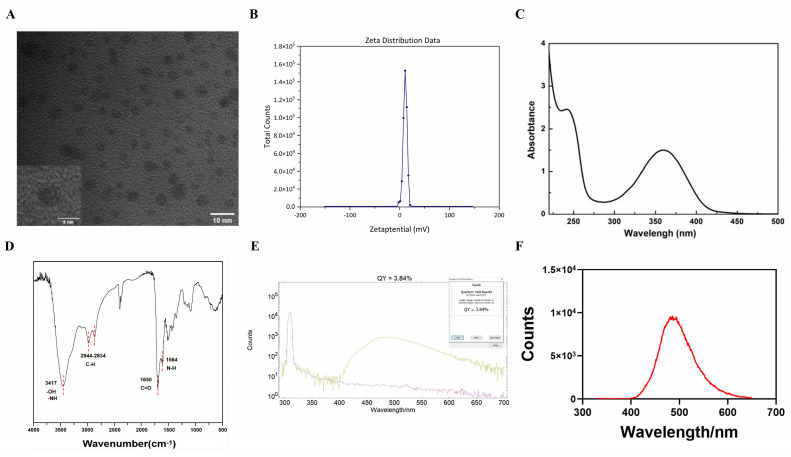
Characterization of the CA-PEI CDs. (**A**) Electron microscopy images. (**B**) potentiogram. (**C**) ultraviolet-visible absorption spectra. (**D**) FTIR map. (**E**) QY value. The yellow line indicates CA-PEI CDs, and the purple line indicates the baseline. (**F**) fluorescence spectra of the CA-PEI CDs.

Polyethyleneimine (PEI)-passivated carbon dots (CA-PEI CDs) exhibit favorable water solubility and appear as a yellowish transparent liquid when dissolved in water. High-resolution transmission electron microscopy revealed that the CA-PEI CDs were predominantly spherical, with a particle size ranging from 3 to 7 nm (Figure 1A). Charge measurements indicate that the CA-PEI CDs possess a positive charge (Figure 1B). Spectral analysis revealed two main absorption bands in the ultraviolet range of 200–600 nm for the CA-PEI CDs (Figure 1C). Notably, absorption peaks at 245 nm and 360 nm are observed, possibly corresponding to the electronic transition of π-π* orbitals within the polycyclic aromatic domain and the n-π* transition in C-N bonds, respectively. The Fourier transform infrared spectroscopy (FTIR) spectrum of the CA-PEI CDs suggested that the absorption peak at 3417 cm^−1^ could be associated with O-H and N-H vibration stretching, whereas those at 2944 cm^−1^ and 2834 cm^-1^ were associated with C-H. The peak at 1650 cm^−1^ originated from C=O stretching vibrations, whereas the peak at 1564 cm^−1^ was associated with N-H vibrations (Figure 1D). When quinine sulfate was used as the standard, the quantum yield (QY) of the CA-PEI CDs was 3.84% (Figure 1E), which is characteristic of fluorescence. Furthermore, when excited at a wavelength of 310 nm, the CA-PEI CDs exhibit an emission wavelength of approximately 485 nm (Figure 1F).

### 2.2. Establishment of the APP/PS1-N2a Cell Line

**Figure 2:**
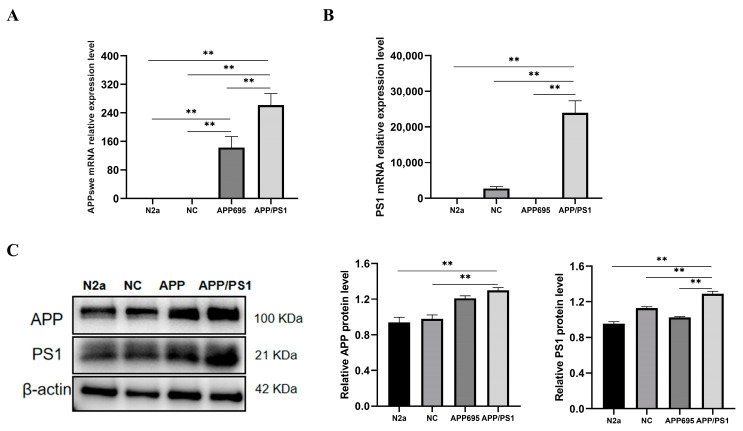
APP and PS1 mRNA and protein levels in APP/PS1-N2a cells. (**A**) APP mRNA levels. (**B**) PS1 mRNA levels. (**C**) APP and PS1 protein levels. The values are presented as the means ± SDs. ** *p* < 0.01 according to one-way ANOVA.

The established plasmid (PS1) was transfected into APP/PS1-N2a cells, and monoclonal cell lines were screened using 10 μg/mL blasticidin and 2 μg/mL puromycin. The selected monoclonal cells were subsequently verified. The efficiency of PS1 expression was determined through qPCR (Figure 2A,B) and Western blotting (Figure 2C). APP levels were significantly greater in both N2a/APP695 cells and APP/PS1-N2a cells than in N2a cells. Furthermore, compared to those in N2a/APP695 cells, PS1 levels in APP/PS1-N2a cells were significantly greater. These results confirmed the successful generation of APP/PS1-N2a cells.

### 2.3. Cytotoxic Effect Assessment of the CA-PEI CDs

**Figure 3:**
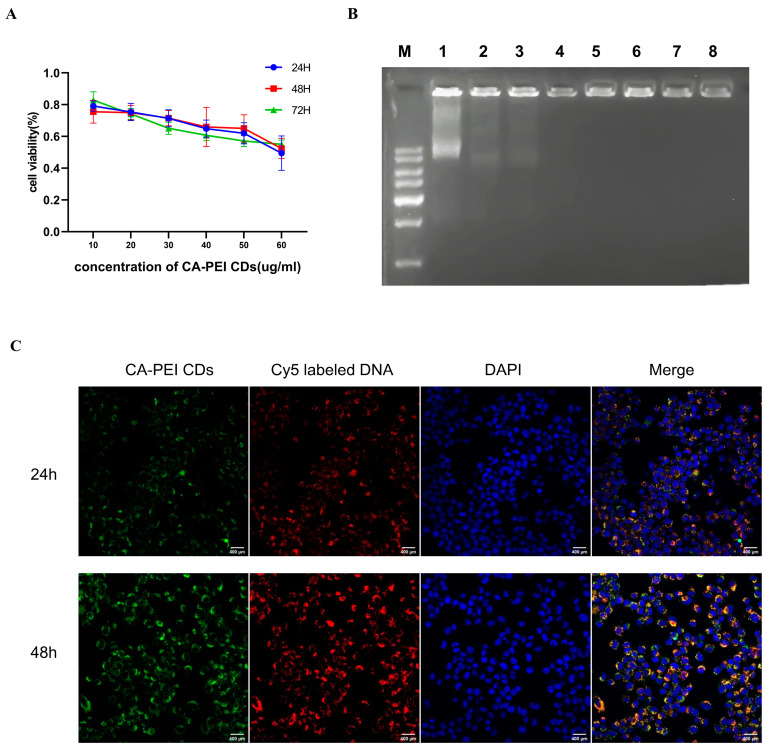
CA-PEI CDs were used as vectors to transfect DNA into APP/PS1-N2a cells. (**A**) An MTT assay was used to detect the toxicity of the CA-PEI CDs (the CA-PEI CD concentration used in the experiment was 4 μg/mL). (**B**) The ability of the CA-PEI CDs to concentrate DNA and the optimal binding ratio were detected by agarose gel electrophoresis. (**C**) Fluorescence images of APP/PS1-N2a cells transfected with CA-PEI CDs-concentrated Cy5-labeled DNA after 24 h and 48 h. Bar = 400 µm.

Biological delivery vectors require CA-PEI CDs to exhibit high transfection efficiency and low cytotoxic effects. To determine the cytotoxic effect of the CA-PEI CDs, an MTT assay was performed to determine the relative survival rate of APP/PS1-N2a cells exposed to the CA-PEI CDs. The cytotoxicity of the CA-PEI CDs occurred in a dose-dependent manner, with no significant time-dependent variation (Figure 3A). This observed cytotoxicity may be due to the abundant positive charge of PEI, leading to severe damage to the cell membrane. The concentration of the CA-PEI CDs utilized in this experiment was 4 μg/mL.

### 2.4. CA-PEI CD/pDNA Complex Formation

The CA-PEI CDs facilitated DNA condensation via electrostatic interactions between the positively charged surface of PEI and the negatively charged phosphate backbone of pDNA. Agarose gel electrophoresis was performed to examine the binding of the CA-PEI CDs to DNA at various complex ratios. The intensity of the DNA migration band within the agarose gel slightly decreased with increasing weight ratio. Ultimately, at a weight ratio of 4, the pDNA was entirely confined to the loading well, signifying the excellent ability of the CA-PEI CDs to concentrate DNA (Figure 3B).

### 2.5. Cell Imaging Study

Due to their biocompatibility, photostability, facile functionalization, excellent photoluminescence, and water solubility, CDs have extensive applications in various biomedical fields, including live cell imaging, electronics, catalysis, targeted drug delivery, and biosensing [24]. To monitor the DNA transfer process, CA-PEI CDs were added to encapsulate Cy5-labeled DNA. Subsequently, the complex was co-incubated with APP/PS1-N2a cells for various durations of transfection. The green signal emitted by the CA-PEI CDs overlapped with the red signal from Cy5-labeled DNA, resulting in a yellow signal within the cytoplasm at 24 h, as shown in the merged view. This suggests the successful delivery of DNA into cells by the CA-PEI CDs. With increasing transfection time, the intracellular independent red signals also gradually increased, indicating the release of DNA from the CA-PEI CDs (Figure 3C). Due to their outstanding fluorescence performance, CA-PEI CDs can track the gene transfection process in real time.

### 2.6. PINK1 Reduced ROS Levels in APP/PS1-N2a Cells

**Figure 4:**
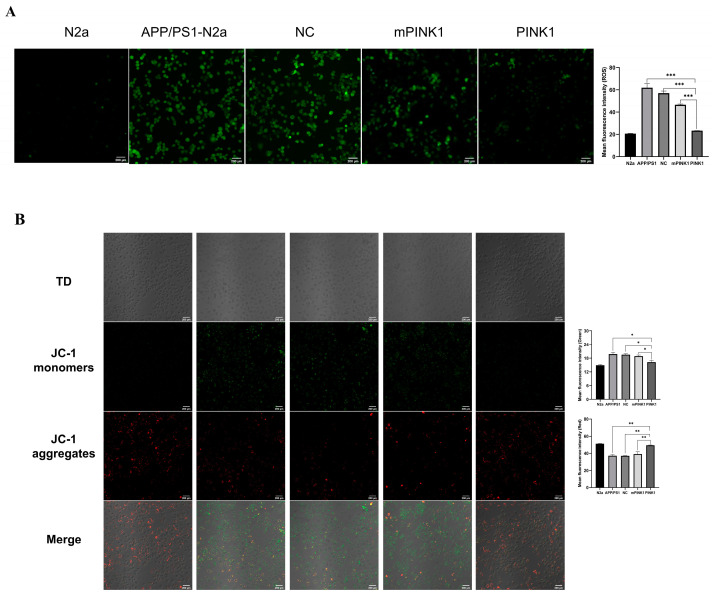
PINK1 improves mitochondrial function. Bar = 200 µm. (**A**) PINK1 reduced ROS production in APP/PS1-N2a cells. (**B**) PINK1 increased the mitochondrial membrane potential in APP/PS1-N2a cells. The values are presented as the means ± SDs. * *p* < 0.05, ** *p* < 0.01, *** *p* < 0.001 according to one-way ANOVA.

PINK1 has a protective effect on APP/PS1-N2a cells. To determine ROS levels in APP/PS1-N2a cells, DCFH-DA was added for cell labeling before observation with a laser scanning confocal microscope. Compared with the APP/PS1-N2a group, the PINK1 group exhibited significantly reduced ROS accumulation in APP/PS1-N2a cells after 24 h of treatment with the CA-PEI CD/pDNA complexes (Figure 4A).

### 2.7. PINK1 Improves Mitochondrial Function by Increasing the MMP and Intracellular ATP Levels

To determine the effect of PINK1 on mitochondrial activity, changes in the MMP were examined. Compared with those in the N2a group, JC-1 in the APP/PS1-N2a group exhibited increased green fluorescence intensity and decreased red fluorescence intensity, indicating a decrease in the MMP. After 24 h of treatment with the CA-PEI CD/pDNA complexes, the PINK1 group exhibited increased red fluorescence intensity and decreased green fluorescence intensity (Figure 4B), suggesting that PINK1 protects against mitochondrial membrane depolarization in APP/PS1-N2a cells.

**Figure 5:**
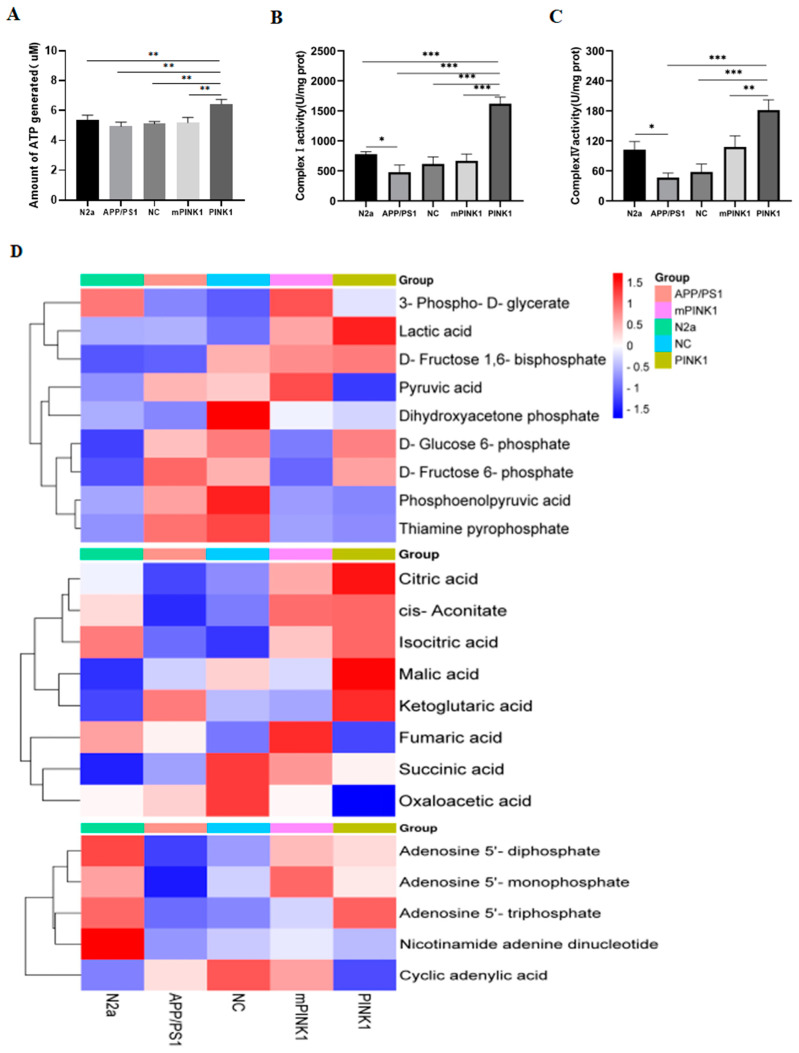
PINK1 improves ATP-related energy metabolism. (**A**) PINK1 increases ATP levels in N2a and APP/PS1-N2a cells. (**B**) PINK1 increases CI activity in N2a and APP/PS1-N2a cells. (**C**) PINK1 increases CIV activity in N2a and APP/PS1-N2a cells. (**D**) PINK1 induces changes in the levels of metabolites related to glycolysis, the tricarboxylic acid cycle, and ATP production in N2a and APP/PS1-N2a cells. The values are presented as the means ± SDs. * *p* < 0.05, ** *p* < 0.01, *** *p* < 0.001 according to one-way ANOVA.

The mitochondrial membrane potential serves as the driving force for ATP production, and a loss of the mitochondrial membrane potential can lead to a reduction in intracellular ATP levels. Therefore, intracellular ATP levels serve as a crucial indicator for determining mitochondrial function. Compared with that in the N2a group, the ATP content in the APP/PS1-N2a group significantly decreased. After 24 h of treatment with the CA-PEI CD/pDNA complexes, the ATP level increased in the PINK1 group (Figure 5A).

### 2.8. PINK1 Induces ATP-Related Metabolic Changes in APP/PS1-N2a Cells

We determined the enzyme activity of NADH ubiquinone oxidoreductase (complex I, CI), succinate ubiquinone oxidoreductase (complex II, CII), ubiquinol cytochrome C oxidoreductase (complex III, CIII), and cytochrome C oxidase (complex IV, CIV) using a micromethod (only positive results are presented here). Compared with those in the N2a group, the enzyme activities of CI and CIV in APP/PS1-N2a cells were significantly impaired, leading to defects in ATP synthesis in these cells. After transfection with CA-PEI CD/pDNA complexes for 24 h, the expression of PINK1 rescued the loss of CI and CIV enzyme activity in APP/PS1-N2a cells (Figure 5B,C), consistent with the changes observed in ATP synthesis.

LC-MS/MS was used to analyze ATP-related metabolites in N2a and APP/PS1-N2a cells. Compared with those in N2a cells, the levels of TCA cycle intermediates (citric acid, aconitic acid, isocitric acid, and fumaric acid) in APP/PS1-N2a cells were significantly lower, whereas the levels of glycolytic intermediates (1,6-diphosphate fructose, β-6-phosphate fructose, D-glucose-6-phosphate, lactic acid, phosphoenolpyruvate, pyruvate, and thiamine pyrophosphate) were greater. The effect in the PINK1 group was opposite to that in the APP/PS1-N2a group. The levels of isocitric acid, citric acid, malic acid, and α-ketoglutaric acid in the TCA cycle increased, whereas the level of oxaloacetic acid decreased. The levels of glycolysis intermediates, phosphoenolpyruvate, and the final product pyruvate decreased. The contents of AMP and ADP, the raw materials for ATP synthesis, decreased, whereas the level of ATP increased. These results led to the conclusion that ATP-related metabolism was significantly increased after 24 h of PINK1 treatment in APP/PS1-N2a cells (Figure 5D).

### 2.9. PINK1 Decreased Aβ1-40 and Aβ1-42 Levels in APP/PS1-N2a Cells

**Figure 6:**
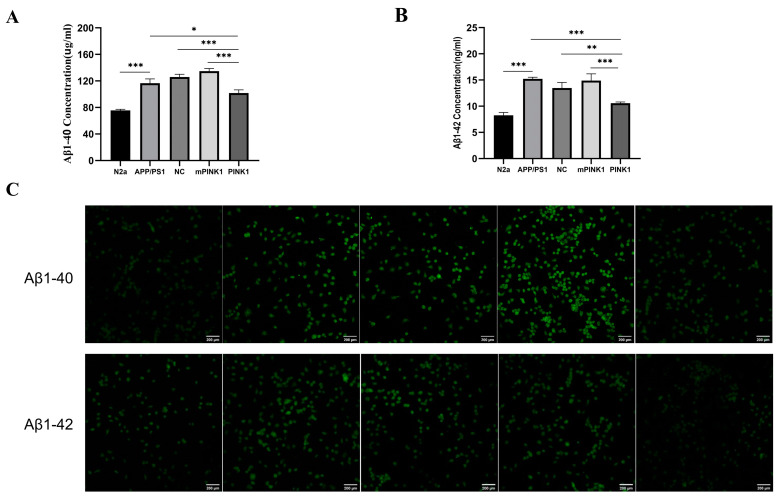
PINK1 decreases Aβ1-40 and Aβ1-42 levels in APP/PS1-N2a cells. Bar = 200 µm. (**A**,**B**) Aβ1-40 and Aβ1-42 expression in N2a and APP/PS1-N2a cells was detected by ELISA. (**C**) Immunofluorescence images of Aβ1-40 and Aβ1-42 in N2a and APP/PS1-N2a cells were captured by laser confocal microscopy. The values are the means ± SDs. * *p* < 0.05, ** *p* < 0.01, *** *p* < 0.001 according to one-way ANOVA.

To determine the effect of PINK1 expression on Aβ1-40 and Aβ1-42 levels, we measured the expression of Aβ1-40 and Aβ1-42 in the culture medium of APP/PS1-N2a cells transfected with CA-PEI CD/pDNA complexes for 24 h using ELISA. Additionally, we detected intracellular Aβ1-40 and Aβ1-42 levels through an immunofluorescence assay. Compared with those in the N2a group, the Aβ1-40 and Aβ1-42 levels in the APP/PS1-N2a group were significantly greater (Figure 6A–C). The expression of PINK1 significantly decreased the Aβ1-40 and Aβ1-42 levels in the APP/PS1-N2a cells (Figure 6A–C).

## 3. Discussion

Cellular energy deficits and mitochondrial dysfunction affect the pathophysiology of aging and AD [25]. Presently, AD is treated via drug therapy. However, it is effective in managing AD symptoms rather than curing or preventing AD [26]. High metabolic activity at the BBB leads to the degradation of most internalized substances [27]. Peripheral drugs cannot cross the BBB; therefore, genes that allow a single, long-lasting intervention in AD are particularly attractive [28]. Viral vectors are used for gene delivery; however, there are still many risks in gene delivery, including triggering immune and inflammatory responses, limiting cloning capacity, and complex production methods [29]. CDs are used as optimal substitutes due to their low toxicity and biocompatibility. CDs prepared with pentaethylenehexamine and citric acid as precursors have been shown to carry plasmids across the blood–brain barrier in zebrafish models [30]. This suggests that CDs have immense potential in gene delivery for AD treatment, which is consistent with our experimental results. In our study, CDs successfully transferred PINK1 into APP/PS1-N2a cells. We found decreased levels of ROS, Aβ1-40, and Aβ1-42 and increased levels of mitochondrial membrane potential and ATP, which may be associated with PINK1 decreasing oxidative stress and improving energy metabolism disorders in APP/PS1-N2a cells. Genetic studies have shown that autosomal dominant AD caused by APP, PS1, and PS2 mutations is associated with abnormal Aβ production [31]. Previous studies have shown that several familial AD mutations in the above three genes lead to a greater Aβ42/Aβ40 ratio, as well as the formation of toxic Aβ42 oligomers and plaques in the brains of patients [32]. Extended Aβ species (Aβ43 and/or Aβ42) and β-CTF aggregates were also found in several APP and PS1 mutation-carrying isogenic knock-in human iPSC lines [33]. Hence, we selected APP/PS1-N2a cells as the AD cell model and observed significant increases in Aβ1-40 and Aβ1-42 in APP/PS1-N2a cells. Bare nucleic acids can be quickly destroyed via serum nucleases. Therefore, to improve gene therapy efficacy, efficient vectors should be prepared for compressing and preserving nucleic acids with low toxicity and a high gene transfection rate [34]. CDs concentrate ctDNA through strong electrostatic interactions, which do not change the DNA structure and allow the slow release of ctDNA via the dialysis membrane within the physiological medium [35]. Therefore, we selected CDs synthesized from citric acid (CA) and PEI as a gene delivery carrier. Our findings indicate that CDs can be condensed with plasmids, which can successfully transfect APP/PS1-N2a cells, taking advantage of the electrostatic interaction between the positive charge on the surface of CDs and the negative charge of the DNA phosphate group [36].

PINK1 is a mitochondrial serine/threonine-protein kinase that is crucial for mitochondrial quality control [37]. Under normal conditions, intracellular PINK1 is cleaved into cPINK1 and released into the cytoplasm. cPINK1 improves the ability of cells to resist stress and activate pro-survival pathways through many pathways. This process regulates mitochondrial biogenesis and inhibits mitophagy [23,38]. In the case of mitochondrial injury, PINK1 maintains mitochondrial homeostasis via the regulation of mitochondrial fission and fusion, the balance between the supply and demand of mitochondrial bioenergy, and the initiation of mitophagy [23,39]. In a tau hyperphosphorylation rat model, the knockout of PINK1 aggravated spatial working memory impairment, synaptic dysfunction, and hippocampal neuronal loss, whereas PINK1 upregulation mitigated tau hyperphosphorylation in rats and reversed aberrant alterations in mitochondrial dynamics, defective mitophagy, and decreased hippocampal ATP levels [40]. Severe mitochondrial damage, mitochondrial ROS, and apoptosis were also detected in PINK1-deficient cells and mice [41]. In this study, we found that PINK1 decreased oxidative stress and increased ATP levels and mitochondrial membrane potential in APP/PS1-N2a cells. This may be because PINK1 overexpression inhibits mitochondrial fusion, improves mitochondrial impairment, and decreases neuroinflammatory cytokine generation [14].

Mitochondrial dysfunction and the accumulation of the Aβ peptide are early pathological characteristics of patients with AD. Furthermore, PINK1 is associated with AD pathology. Gene therapy-mediated PINK1 overexpression decreased Aβ expression, amyloid-related pathology, mitochondrial and synaptic dysfunction, and oxidative stress in mPINK1 mice; improved autophagy signals by activating autophagy receptors (OPTN and NDP52); and increased the clearance of damaged mitochondria [13]. The PINK1-dependent pathway activates mitophagy, increases the clearance of extracellular Aβ plaques, inhibits neuroinflammation, relieves tau hyperphosphorylation, and reverses memory impairment [42]. Taken together, these results indicate that PINK1 can decrease the pathology and symptoms of AD through mitophagy. However, the relationship between PINK1 and the mitochondrial energy supply in AD is still unknown. Therefore, we further determined the effects of PINK1 on glycolysis, the TCA cycle, and the mitochondrial respiratory chain.

Aberrant intracerebral energy metabolism in patients with AD occurs before the onset of clinical symptoms [43,44]. Glucose undergoes glycolysis to produce reducing equivalents of ATP and NADH. Furthermore, the end-product pyruvate feeds into the mitochondrial TCA cycle [45]. The high-energy electrons produced by the TCA are delivered downward to the electron transport chain (ETC), resulting in a proton gradient produced by ATP [46,47]. The overexpression of PINK1 in mouse colon cancer cells can increase mitophagy, decrease glycolysis, and increase mitochondrial respiration by activating the p53 pathway [48]. These findings suggest that PINK1 is closely associated with cell energy metabolism and that increased energy metabolism can improve AD-related pathological changes. Therefore, we determined the changes in ATP-related metabolite levels by LC-MS/MS. We found that the accumulation of pyruvate, the final product of glycolysis, decreased. Under normal circumstances, pyruvate enters the mitochondria and is decarboxylated by the pyruvate dehydrogenase complex to acetyl-CoA [49]. Acetyl-CoA and oxaloacetate are substrates of citrate synthase, which synthesizes citric acid to start the TCA cycle [49,50]. Therefore, the reduced oxaloacetate and pyruvate levels and increased citric acid levels may be associated with the increase in citric acid synthase activity [51]. Citrate and acetyl-CoA deposition suppresses 6-phosphofructokinase 1 and pyruvate kinase, respectively, slowing glycolysis and preventing pyruvate deposition [52]. When citric acid levels are increased, high levels of α-ketoglutarate and succinic acid are associated with improved isocitrate dehydrogenase and α-ketoglutarate dehydrogenase complex activities. In patients with AD, the activities of mitochondrial enzymes associated with energy generation, including α-ketoglutarate dehydrogenase, citrate synthase isocitrate dehydrogenase, CIV, and ATP synthase, decrease, whereas the activities of succinate dehydrogenase and malate dehydrogenase increase [51]. We detected the level of the mitochondrial TCA complex and found that the enzyme activity of CI and CIV increased. Mitochondrial respiration resulting from the ETC was significantly decreased within mitochondria isolated from PINK1-KO Drosophila, which decreased ETC CI and CIV enzyme activity. PINK1-KO drosophila exhibits defects in mitochondrial fission [53]. Hence, PINK1 can increase defects in the assembly of ETC complexes by increasing mitochondrial fission to reverse mitochondrial energy metabolism disorders. PINK1 overexpression in APP/PS1-N2a cells increased the TCA cycle and OXPHOS, and compensatory glycolysis decreased.

Extracellular senile plaque is a unique pathological characteristic of AD. Furthermore, Aβ is the main component of senile plaque [54]. APP can be abnormally cleaved via β-secretase and γ-secretase within the extracellular space to produce Aβ1-40 and Aβ1-42 [55]. Hence, we evaluated the levels of Aβ1-40 and Aβ1-42 in APP/PS1-N2a cells after transfection with PINK1 for 24 h. The findings revealed that PINK1 overexpression in APP/PS1-N2a cells led to a reduction in Aβ1-40 and Aβ1-42 levels. These findings indicate that PINK1 has the potential to alleviate AD-related pathology by improving mitochondrial energy metabolism.

## 4. Materials and Methods

### 4.1. Materials

CA and PEI were obtained from Shanghai Yuanye Biotechnology Co., Ltd. (Shanghai, China). Plasmids containing PINK1 (H23964), mPINK1 (H23965), and PS1 (H25228) were obtained from Shanghai Heyuan Biotechnology Co., Ltd. (Shanghai, China). Rabbit anti-Aβ1-40 (bs-0106 M) and rabbit anti-Aβ1-42 (bs-0107R) were purchased from Bioss (Beijing, China). APP rabbit anti-mouse IgG and PS1 rabbit anti-mouse IgG were obtained from Proteintech (Wuhan, China). HRP-conjugated goat anti-mouse and anti-rabbit IgG were obtained from Biosharp (Anhui, China).

### 4.2. Preparation of CA–PEI CDs

With CA and PEI as the raw materials, CA-PEI CDs were prepared through a hydrothermal approach. The obtained solution was subsequently added to an autoclave at 180 °C for 6 h following which 0.1 g of CA and 0.2 g of PEI were dissolved in 20 mL of deionized water. After the reaction, the solution was centrifuged for 15 min at 5000 rpm to collect the supernatant in a dialysis bag (1000 kDa). The liquid obtained after dialysis was freeze-dried, and the powder obtained was CA-PEI CDs.

### 4.3. Instrumentation and Characterization

Morphological characterization of the CA-PEI CDs was conducted using high-resolution transmission electron microscopy (HRTEM) on an HT7800 microscope (Hitachi, Tokyo, Japan). A UV-2600i ultraviolet-visible spectrophotometer (Shimadzu, Kyoto, Japan) was used to characterize its UV-vis absorption. FTIR analysis was performed using a Frontier spectrometer (PerkinElmer, Waltham, MA, USA). Photoluminescence (PL) emission measurements were carried out using an FS5 fluorescence meter (Edinburgh, Livingston, LA, England). The ζ potential of the CA-PEI CDs was determined with a Zetasizer Nano ZSE potential analyzer (Malvern Panalytical, Shanghai, China). 

### 4.4. Measurement of Fluorescence QY

The QY of the CA-PEI CDs was determined using quinine sulfate as the standard and calculated according to the following equation:

(1) $$Q=Q_{r}\times \frac{I}{I_{r}}\times \frac{A_{r}}{A}\times {\left(\frac{n}{n_{r}}\right)}^{2}$$

where *Q* represents the QY, *I* represents the measured laser emission intensity, *n* indicates the solvent refractive index, and *A* is the optical density.

### 4.5. Agarose Gel Retardation Assay

PINK1 was used as the model DNA. A total of 10 μL of PINK1 (1.095 mg/mL) was mixed with various material volumes to prepare material/DNA complexes at different mass ratios (*w*/*w*). Subsequently, these complexes were subjected to 50 min of electrophoresis on a 1% (*w*/*v*) agarose gel containing GelRed in tris-acetate-EDTA (TAE) running buffer at 100 V. Following electrophoresis, an automatic chemiluminescence image analysis system (ABLX5; Tanon, Shanghai, China) was used to visualize the DNA under ultraviolet light.

### 4.6. Cell Culture

We obtained N2a cells from the Chinese Academy of Sciences, and N2a/APP695 cells were provided by Professor Xu Huaxi from Xiamen University. N2a mouse neuroblastoma cells were cultured in MEM, while N2a/APP695 mouse neuroblastoma cells were cultured in Opti-MEM and DMEM F12. Both media were supplemented with 10% FBS and 1% penicillin-streptomycin. Additionally, 1% nonessential amino acid (NEAA) solution was added to the N2a cell medium, and 200 μL of Geneticin^™^ selective antibiotic solution was added to the N2a/APP695 cell medium. The cells were incubated in an environment with 5% CO_2_ and 95% humidity.

### 4.7. PS1 Gene-Transfected N2a/APP695 Cells

To establish N2a cells overexpressing the APP and PS1 genes, the PS1 gene was transfected into N2a/APP695 cells. The PS1 gene was obtained through polymerase chain reaction (PCR) amplification, and the resulting target gene was ligated to the linearized vector using DNA ligase to generate the PS1 plasmid. The PS1 plasmid was then transfected into N2a/APP695 cells via lentivirus, and the cells obtained through screening with blasticidin (10 μg/mL) and puromycin (2 μg/mL) were designated APP/PS1-N2a cells.

### 4.8. mRNA Quantification Analysis (qRT-PCR)

APP and PS1 mRNA expression in APP/PS1-N2a cells was observed. A total RNA extraction kit (Promega, Shanghai, China) was used for extracting total RNA. First-strand premix reagent (Tiangen, Beijing, China) for one-step genomic cDNA synthesis was used to synthesize cDNA, and SuperReal fluorescence quantitative premix reagent (Tiangen, Beijing, China) was used for real-time fluorescence quantitative PCR, with β-actin serving as the endogenous control. The following qPCR primers were used:

*APP* forward: GTTTGGCACTGCTCCTGCTGT; reverse: ACATGGCAATCTGGGGTTCA; *PS1* forward: GACAACCACCTGAGCAATACTGT; reverse: CTCATCTTGCTCCACCACCTG; *β-actin* forward: GGGTGTGATGGTGGGAATGG; and reverse: GGTTGGCCTTAGGGTTCAGG.

### 4.9. Western Blot Analysis

The protein expression of the APP and PS1 genes in APP/PS1-N2a cells was quantitatively analyzed through Western blotting. A Bradford protein assay (BSA) was used to determine the protein concentration. A total of 15 μg of extracted protein was loaded onto a 10% SDS-PAGE gel for electrophoresis. Subsequently, the protein bands were transferred to a 0.45 μm nitrocellulose membrane using a membrane transfer instrument (PowerPac^TM^, Bio-Rad, Hercules, CA, USA). The expression of the APP and PS1 proteins was detected using rabbit polyclonal antibodies against APP and PA1, respectively. The protein level of β-actin, which served as an internal control, was determined using a rabbit polyclonal antibody against β-actin.

### 4.10. Cell Viability Assay

An MTT assay was performed to evaluate the cytotoxic effect of the CA-PEI CDs. APP/PS1-N2a cells (5 × 10^4^/well) were inoculated into 96-well plates and incubated overnight. On the subsequent morning, 10, 20, 30, 40, 50, and 60 μg/mL CA-PEI CDs were added for cell treatment for 24, 48, and 72 h, after which the CA-PEI CD-containing medium was discarded, followed by the addition of 100 μL fresh medium that contained 10 μL MTT (5 mg/mL) for 4 h. Finally, all the medium was discarded, and 100 μL/well DMSO was added for 4 h of incubation at 37 °C. The absorbance in each well was measured at 570 nm with a multifunctional microplate detector (BioTek SynergyHTX, Agilent, Santa Clara, CA, USA). Untreated cells (DMEM) served as controls, and relative cell viability (average % ± SD, *n* = 4) was calculated as Abs_sample_/Abs_control_ × 100%.

### 4.11. In Vitro Fluorescence Imaging

Following the manufacturer’s instructions, pDNA was labeled using a Label IT Cy5 labeling kit (Mirus, Madison, WI, USA). In brief, APP/PS1-N2a cells (5 × 10^4^/well) were seeded into a 35 mm confocal culture dish. After 24 h, the CA-PEI CDs (5 μL, 5 mg/mL) were incubated with Cy5-labeled DNA (0.1 mg DNA/well, the optimal sample mass ratio) in an incubator at 37 °C for 30 min. The resulting complex was added to the cell culture medium and incubated for an additional 24 h at 37 °C. Subsequently, the cells were washed with 1 mL of PBS, and fluorescence images were captured using a laser scanning confocal microscope (FV3000, Olympus, Tokyo, Japan).

### 4.12. Intracellular ROS Measurement

ROS levels were evaluated using the nonfluorescent probe dichlorofluorescein diacetate (DCFH-DA; S0033M, Beyotime, Shanghai, China). The cells were incubated with DCFH-DA for 30 min at 37 °C, and subsequently, a laser scanning confocal microscope (FV3000, Olympus, Tokyo, Japan) was used to detect the distribution of DCF fluorescence at excitation and emission wavelengths of 488 and 535 nm, respectively.

### 4.13. MMP

The MMP was determined using the fluorescence dye 5,5′,6,6′-tetrachloro-1,1′,3,3′-tetraethylbenzimidazole-carbocyanine iodide (JC-1; C2003S, Beyotime, Shanghai, China). Following a 30 min incubation with 5 μg/mL JC-1 at 37 °C, the cells were observed using a laser scanning confocal microscope (FV3000, Olympus, Tokyo, Japan). Red fluorescence intensities were measured at excitation and emission wavelengths of 488 and 595 nm, respectively, while green fluorescence intensities were measured at 488 and 525 nm. In the presence of a low MMP, JC-1 exists as a monomer and exhibits green fluorescence, while under a high MMP, it forms polymers and emits red fluorescence.

### 4.14. ATP Assay

ATP levels were measured according to the manufacturer’s protocols (S0026, Beyotime, Shanghai, China), and the fluorescence intensities of different wells were detected using a multifunctional microplate detector (BioTek SynergyHTX, Agilent, Santa Clara, CA, USA). Samples were collected 24 h after the transfection of CA-PEI CD/pDNA complexes into APP/PS1-N2a cells.

### 4.15. Determination of Mitochondrial CI-IV Activity

We used a mitochondrial respiratory chain complex activity assay kit (BC0515, BC3235, BC3245, BC0945; Solarbio, Beijing, China) to detect the activities of CI, CII, CIII, and CIV in cells. A total of 5 × 106 cells were collected, homogenized, and centrifuged to collect the supernatants in a centrifuge tube. The sample was then centrifuged, and the resulting precipitate was resuspended in the extract and sonicated for disruption. The activity of the obtained liquid was determined according to specific protocols. The OD values of CI, CII, CIII, and CIV were measured by a multifunctional microplate detector (BioTek SynergyHTX, Agilent, Santa Clara, CA, USA) at 340 nm, 605 nm, 550 nm, and 550 nm.

### 4.16. LC-MS/MS Analysis

The samples were pre-extracted and redissolved in acetonitrile (A955-4, Fisher Chemical, Waltham, MA, USA) and eluted using two solvent buffers (A: 98.8% ammonium acetate (50 mM) and 1.2% ammonium hydroxide; B: 99% acetonitrile and 1% acetylacetone). The samples were separated by an ultrahigh-performance liquid chromatography system (1290 Infinity UPLC, Agilent, Santa Clara, CA, USA), followed by analysis with a 5500 QTRAP mass spectrometer (SCIEX, Shanghai, China) in negative-ion mode. MultiQuant software (3.0.2) was used to determine the chromatographic peak area and retention time for metabolite identification. Metabolite concentrations were normalized to succinic acid-d6 and total protein levels. A significance level of *p* < 0.05 was considered to indicate statistical significance. A heatmap was generated using https://www.bioinformatics.com.cn (last accessed on 30 January 2024), an online tool used for data analysis and visualization.

### 4.17. Immunofluorescence Assay and Confocal Microscopy

APP/PS1-N2a cells were transfected with the CA-PEI CD/pDNA complexes for 24 h. Afterward, the cells were fixed for 15 min in 4% paraformaldehyde. Subsequently, they were treated with 0.2% Triton X-100 at room temperature for 15 min and then closed for a 1 h period at ambient temperature. After overnight incubation at 4 °C in solutions containing Aβ1-40 or Aβ1-42 antibodies, Alexa Fluor 488 was used to visualize the Aβ1-40 and Aβ1-42 proteins. Nuclear staining was performed using H33342 solution. Fluorescence observation was conducted using a laser-scanning confocal microscope (FV3000, Olympus, Tokyo, Japan).

### 4.18. Quantification of Aβ1-40 and Aβ1-42 by ELISA

An ELISA kit (Meibiao Biotechnology, Jiangsu, China) was used to detect mouse β-amyloid protein (Aβ1-40 and Aβ1-42) in the cell culture supernatant following a 24 h transfection period with CA-PEI CD/pDNA complexes. The assay was conducted according to specific protocols, and after the substrate reaction, the color intensity was measured at 450 nm using a multifunctional microplate detector (BioTek SynergyHTX, Agilent, Santa Clara, CA, USA). The amount of Aβ1-40 or Aβ1-42 was proportional to the color intensity, and the sample results were directly determined using the standard curve.

### 4.19. Statistical Analysis

The results are expressed as the mean ± standard deviation, and each assay was conducted independently in triplicate. The gray values of the Western blotting bands and the fluorescence intensities of the fluorescence images were analyzed using ImageJ software v.1.54. The experimental results were analyzed using GraphPad Prism 9.3.1471, and statistical analysis was performed through a one-way analysis of variance and Tukey’s multiple comparison test. A *p*-value < 0.05 was considered to indicate statistical significance.

## 5. Conclusions

In conclusion, our study showed that CDs exhibit a remarkable ability to concentrate plasmids and facilitate their transfection into cells, suggesting that CDs are a novel choice for selecting gene delivery vectors. Furthermore, our investigation revealed that PINK1 overexpression has the potential to ameliorate cellular energy metabolism disorders, restore mitochondrial function, and decrease the production of Aβ. This opens up a new possibility for regulating the quality and homeostasis of mitochondria. The investigation of ETC complex activity and the analysis of ATP-related metabolomics also offer novel insights into metabolic targets in AD.

## Data Availability

The datasets used and/or analyzed during the current study are available from the corresponding author upon reasonable request.

## References

[1] Sun B.L., Li W.W., Zhu C., Jin W.S., Zeng F., Liu Y.H., Bu X.L., Zhu J., Yao X.Q., Wang Y.J. (2018). Clinical research on Alzheimer’s disease: Progress and perspectives. Neurosci. Bull..

[2] Li Y., Xia X., Wang Y., Zheng J.C. (2022). Mitochondrial dysfunction in microglia: A novel perspective for pathogenesis of Alzheimer’s disease. J. Neuroinflammation.

[3] Nunnari J., Suomalainen A. (2012). Mitochondria: In sickness and in health. Cell.

[4] Jiang Y., Huo S., Mizuhara T., Das R., Lee Y.W., Hou S., Moyano D.F., Duncan B., Liang X.J., Rotello V.M. (2015). The interplay of size and surface functionality on the cellular uptake of sub-10 nm gold nanoparticles. ACS Nano.

[5] Dhillon S. (2021). Aducanumab: First approval. Drugs.

[6] Hoy S.M. (2023). Lecanemab: First approval. Drugs.

[7] Shi M., Chu F., Zhu F., Zhu J. (2022). Impact of anti-amyloid-beta monoclonal antibodies on the pathology and clinical profile of alz-heimer’s disease: A focus on aducanumab and lecanemab. Front. Aging Neurosci..

[8] Lin M.T., Beal M.F. (2006). Mitochondrial dysfunction and oxidative stress in neurodegenerative diseases. Nature.

[9] Shoshan-Barmatz V., Nahon-Crystal E., Shteinfer-Kuzmine A., Gupta R. (2018). VDAC1, mitochondrial dysfunction, and Alzheimer’s disease. Pharmacol. Res..

[10] Dagda R.K., Pien I., Wang R., Zhu J., Wang K.Z., Callio J., Banerjee T.D., Dagda R.Y., Chu C.T. (2014). Beyond the mitochondrion: Cytosolic PINK1 remodels dendrites through protein kinase A. J. Neurochem..

[11] Jiang X., Jin T., Zhang H., Miao J., Zhao X., Su Y., Zhang Y. (2019). Current progress of mitochondrial quality control pathways underlying the pathogenesis of parkinson’s disease. Oxidative Med. Cell. Longev..

[12] Han Y., Liu D., Cheng Y., Ji Q., Liu M., Zhang B., Zhou S. (2023). Maintenance of mitochondrial homeostasis for Alzheimer’s disease: Strategies and challenges. Redox Biol..

[13] Du F., Yu Q., Yan S., Hu G., Lue L.F., Walker D.G., Wu L., Yan S.F., Tieu K., Yan S.S. (2017). PINK1 signalling rescues amyloid pathology and mitochondrial dysfunction in Alzheimer’s disease. Brain.

[14] Wang X., Xue Y., Yao Y., Li Y., Ji X., Chi T., Liu P., Zou L. (2022). PINK1 regulates mitochondrial fission/fusion and neuroinflammation in β-amyloid-induced Alzheimer’s disease models. Neurochem. Int..

[15] Patro S., Ratna S., Yamamoto H.A., Ebenezer A.T., Ferguson D.S., Kaur A., McIntyre B.C., Snow R., Solesio M.E. (2021). Atp synthase and mitochondrial bioenergetics dysfunction in Alzheimer’s disease. Int. J. Mol. Sci..

[16] Butterfield D.A., Halliwell B. (2019). Oxidative stress, dysfunctional glucose metabolism and Alzheimer disease. Nat. Rev. Neurosci..

[17] Ardanaz C.G., Ramírez M.J., Solas M. (2022). Brain metabolic alterations in Alzheimer’s disease. Int. J. Mol. Sci..

[18] Cunnane S.C., Trushina E., Morland C., Prigione A., Casadesus G., Andrews Z.B., Beal M.F., Bergersen L.H., Brinton R.D., de la Monte S. (2020). Brain energy rescue: An emerging therapeutic concept for neurodegenerative disorders of ageing. Nat. Rev. Drug Discov..

[19] Zhang W., Sigdel G., Mintz K.J., Seven E.S., Zhou Y., Wang C., Leblanc R.M. (2021). Carbon dots: A future blood-brain barrier penetrating nanomedicine and drug nanocarrier. Int. J. Nanomed..

[20] Lamptey R.N.L., Chaulagain B., Trivedi R., Gothwal A., Layek B., Singh J. (2022). A review of the common neurodegenerative disorders: Current therapeutic approaches and the potential role of nanotherapeutics. Int. J. Mol. Sci..

[21] Seven E.S., Seven Y.B., Zhou Y., Poudel-Sharma S., Diaz-Rucco J.J., Kirbas Cilingir E., Mitchell G.S., Van Dyken J.D., Leblanc R.M. (2021). Crossing the blood-brain barrier with carbon dots: Uptake mechanism and *in vivo* cargo delivery. Nanoscale Adv..

[22] Zhang W., Kandel N., Zhou Y., Smith N., Ferreira B.C., Perez M., Claure M.L., Mintz K.J., Wang C., Leblanc R.M. (2022). Drug delivery of memantine with carbon dots for Alzheimer’s disease: Blood-brain barrier penetration and inhibition of tau aggregation. J. Colloid Interface Sci..

[23] Wang N., Zhu P., Huang R., Wang C., Sun L., Lan B., He Y., Zhao H., Gao Y. (2020). PINK1: The guard of mitochondria. Life Sci..

[24] Boakye-Yiadom K.O., Kesse S., Opoku-Damoah Y., Filli M.S., Aquib M., Joelle M.M.B., Farooq M.A., Mavlyanova R., Raza F., Bavi R. (2019). Carbon dots: Applications in bioimaging and theranostics. Int. J. Pharm..

[25] Song T., Song X., Zhu C., Patrick R., Skurla M., Santangelo I., Green M., Harper D., Ren B., Forester B.P. (2021). Mitochondrial dysfunction, oxidative stress, neuroinflammation, and metabolic alterations in the progression of Alzheimer’s disease: A meta-analysis of in vivo magnetic resonance spectroscopy studies. Ageing Res. Rev..

[26] Breijyeh Z., Karaman R. (2020). Comprehensive review on Alzheimer’s disease: Causes and treatment. Molecules.

[27] Zhong G., Long H., Zhou T., Liu Y., Zhao J., Han J., Yang X., Yu Y., Chen F., Shi S. (2022). Blood-brain barrier Permeable nanoparticles for Alzheimer’s disease treatment by selective mitophagy of microglia. Biomaterials.

[28] Sun J., Roy S. (2021). Gene-based therapies for neurodegenerative diseases. Nat. Neurosci..

[29] Butt M.H., Zaman M., Ahmad A., Khan R., Mallhi T.H., Hasan M.M., Khan Y.H., Hafeez S., Massoud E.E.S., Rahman M.H. (2022). Appraisal for the potential of viral and nonviral vectors in gene therapy: A review. Genes.

[30] Zhang W., Chen J., Gu J., Bartoli M., Domena J.B., Zhou Y., Ferreira B.C., Kirbas Cilingir E., McGee C.M., Sampson R. (2023). Nano-carrier for gene delivery and bioimaging based on pentaetheylenehexamine modified carbon dots. J. Colloid Interface Sci..

[31] Brase L., You S.F., D’Oliveira Albanus R., Del-Aguila J.L., Dai Y., Novotny B.C., Soriano-Tarraga C., Dykstra T., Fernandez M.V., Budde J.P. (2023). Single-nucleus RNA-sequencing of autosomal dominant Alzheimer disease and risk variant carriers. Nat. Commun..

[32] Kim M., Bezprozvanny I. (2021). Conformational models of APP processing by gamma secretase based on analysis of pathogenic mutations. Int. J. Mol. Sci..

[33] Kwart D., Gregg A., Scheckel C., Murphy E.A., Paquet D., Duffield M., Fak J., Olsen O., Darnell R.B., Tessier-Lavigne M. (2019). A large panel of isogenic APP and PSEN1 mutant human iPSC neurons reveals shared endosomal abnormalities mediated by APP β-CTFs, not Aβ. Neuron.

[34] Guo F., Li Q., Zhang X., Liu Y., Jiang J., Cheng S., Yu S., Zhang X., Liu F., Li Y. (2022). Applications of carbon dots for the treatment of Alzheimer’s disease. Int. J. Nanomed..

[35] Massaro M., Barone G., Biddeci G., Cavallaro G., Di Blasi F., Lazzara G., Nicotra G., Spinella C., Spinelli G., Riela S. (2019). Halloysite nanotubes-carbon dots hybrids multifunctional nanocarrier with positive cell target ability as a potential non-viral vector for oral gene therapy. J. Colloid Interface Sci..

[36] Mohammadinejad R., Dadashzadeh A., Moghassemi S., Ashrafizadeh M., Dehshahri A., Pardakhty A., Sassan H., Sohrevardi S.M., Mandegary A. (2019). Shedding light on gene therapy: Carbon dots for the minimally invasive image-guided delivery of plasmids and noncoding RNAs—A review. J. Adv. Res..

[37] Lee S.Y., An H.J., Kim J.M., Sung M.J., Kim D.K., Kim H.K., Oh J., Jeong H.Y., Lee Y.H., Yang T. (2021). PINK1 deficiency impairs osteoblast differentiation through aberrant mitochondrial homeostasis. Stem Cell Res. Ther..

[38] Soman S.K., Dagda R.K. (2021). Role of cleaved PINK1 in neuronal development, synaptogenesis, and plasticity: Implications for Parkinson’s disease. Front. Neurosci..

[39] Li J., Yang D., Li Z., Zhao M., Wang D., Sun Z., Wen P., Dai Y., Gou F., Ji Y. (2023). PINK1/Parkin-mediated mitophagy in neurodegenerative diseases. Ageing Res. Rev..

[40] Wang X.J., Qi L., Cheng Y.F., Ji X.F., Chi T.Y., Liu P., Zou L.B. (2022). PINK1 overexpression prevents forskolin-induced tau hyperphosphorylation and oxidative stress in a rat model of Alzheimer’s disease. Acta Pharmacol. Sin..

[41] Lin Q., Li S., Jiang N., Shao X., Zhang M., Jin H., Zhang Z., Shen J., Zhou Y., Zhou W. (2019). PINK1-parkin pathway of mitophagy protects against contrast-induced acute kidney injury via decreasing mitochondrial ROS and NLRP3 inflammasome activation. Redox Biol..

[42] Fang E.F., Hou Y., Palikaras K., Adriaanse B.A., Kerr J.S., Yang B., Lautrup S., Hasan-Olive M.M., Caponio D., Dan X. (2019). Mitophagy inhibits amyloid-β and tau pathology and reverses cognitive deficits in models of Alzheimer’s disease. Nat. Neurosci..

[43] Yu L., Jin J., Xu Y., Zhu X. (2022). Aberrant energy metabolism in Alzheimer’s disease. J. Transl. Intern. Med..

[44] Wang W., Zhao F., Ma X., Perry G., Zhu X. (2020). Mitochondria dysfunction in the pathogenesis of Alzheimer’s disease: Recent advances. Mol. Neurodegener..

[45] Tang B.L. (2020). Glucose, glycolysis, and neurodegenerative diseases. J. Cell. Physiol..

[46] Nolfi-Donegan D., Braganza A., Shiva S. (2020). Mitochondrial electron transport chain: Oxidative phosphorylation, oxidant production, and methods of measurement. Redox Biol..

[47] Han S., Zhang M., Jeong Y.Y., Margolis D.J., Cai Q. (2021). The role of mitophagy in the regulation of mitochondrial energetic status in neurons. Autophagy.

[48] Yin K., Lee J., Liu Z., Kim H., Martin D.R., Wu D., Liu M., Xue X. (2021). Mitophagy protein PINK1 suppresses colon tumor growth by metabolic reprogramming via p53 activation and reducing acetyl-CoA production. Cell Death Differ..

[49] Hansen G.E., Gibson G.E. (2022). The alpha-ketoglutarate dehydrogenase complex as a hub of plasticity in neurodegeneration and regeneration. Int. J. Mol. Sci..

[50] Arnold P.K., Finley L.W.S. (2023). Regulation and function of the mammalian tricarboxylic acid cycle. J. Biol. Chem..

[51] Chhimpa N., Singh N., Puri N., Kayath H.P. (2023). The novel role of mitochondrial citrate synthase and citrate in the pathophysiology of Alzheimer’s disease. J. Alzheimers Dis..

[52] Chandel N.S. (2021). Glycolysis. Cold Spring Harb. Perspect. Biol..

[53] Liu W., Acín-Peréz R., Geghman K.D., Manfredi G., Lu B., Li C. (2011). Pink1 regulates the oxidative phosphorylation machinery via mitochondrial fission. Proc. Natl. Acad. Sci. USA.

[54] Zhang Y., Chen H., Li R., Sterling K., Song W. (2023). Amyloid β-based therapy for Alzheimer’s disease: Challenges, successes and future. Signal Transduct. Target. Ther..

[55] Guo T., Zhang D., Zeng Y., Huang T.Y., Xu H., Zhao Y. (2020). Molecular and cellular mechanisms underlying the pathogenesis of Alzheimer’s disease. Mol. Neurodegener..

